# Year-Round Influenza a Virus Surveillance in Mallards (*Anas platyrhynchos*) Reveals Genetic Persistence During the Under-Sampled Spring Season

**DOI:** 10.3390/v12060632

**Published:** 2020-06-11

**Authors:** Sarah E. Lauterbach, Dillon S. McBride, Brendan T. Shirkey, Jacqueline M. Nolting, Andrew S. Bowman

**Affiliations:** 1 Department of Veterinary Preventive Medicine, College of Veterinary Medicine, The Ohio State University, Columbus, OH 43210, USA; lauterbach.7@osu.edu (S.E.L.); mcbride.338@osu.edu (D.S.M.); nolting.4@osu.edu (J.M.N.); 2 Winous Point Marsh Conservancy, Port Clinton, OH 43452, USA; brendan@winous.org

**Keywords:** *Anas platyrhynchos*, influenza A virus, mallards, surveillance, spring migration, phylogenetics

## Abstract

Active influenza A virus (IAV) surveillance in wild waterfowl in the United States has revolved around convenience-based sampling methods, resulting in gaps in surveillance during the spring season. We conducted active IAV surveillance in mallards continuously from July 2017 to July 2019 in the coastal marshes of Lake Erie near Port Clinton, Ohio. We aimed to understand ecological and evolutionary dynamics of IAV across multiple seasons, including the under-sampled spring season. We collected 2096 cloacal swabs and estimated a 6.1% (95% confidence interval (CI): 0.050–0.071) prevalence during the study period. Prevalence was lowest during spring (1.0%, 95% CI: 0.004–0.015). Time-stamped phylogenetic analyses revealed local persistence of genetic lineages of multiple gene segments. The PA segment consists of a lineage detected in multiple seasons with a time to most recent common ancestor of 2.48 years (95% highest posterior density: 2.16–2.74). Analysis of the H3 and H6 segments showed close relation between IAVs detected in spring and the following autumn migration. Though the mechanisms behind viral persistence in a single location are not well understood, we provide evidence that viruses can persist across several seasons. Current surveillance methods should be evaluated to ensure they are capturing the breadth of genetic diversity of IAV in waterfowl and prepare for IAV outbreaks in both animals and humans.

## 1. Introduction

Wild waterfowl, particularly of the orders Anseriformes (ducks, geese, swans) and Charadriiformes (shorebirds, gulls, terns), are considered the primary reservoir of influenza A virus (IAV) [1]. These orders host the largest amount of genetic variation of IAV, including 16 of the 18 known hemagglutinin and nine of the 11 known neuraminidase subtypes [1,2]. Wild waterfowl are generally host to low pathogenic IAVs and show little to no clinical signs of the disease. However, IAVs originating from waterfowl have been involved in devastating highly pathogenic avian influenza outbreaks in commercial poultry and all of the past documented IAV human pandemics [3,4,5]. As birds with long-distance annual migrations, wild waterfowl are implicated in the transmission and spread of IAVs over expansive geographic regions [6,7]. Annual waterfowl migrations play an integral role in the spread of IAVs that may be progenitors of IAVs that infect other species [8]. To understand the threat of IAVs to animal and human health, it is imperative to understand the natural history of IAVs infecting waterfowl populations. Continued active IAV surveillance in wild waterfowl is essential to improve our knowledge and understanding of the epidemiology and ecology of the virus in its primary reservoir.

Active IAV surveillance in wild waterfowl has occurred since their establishment as viable hosts for the virus [9]. The majority of surveillance in North America has been conducted during summer pre-migration and autumn migration, when birds are readily available for sample collection [2,10]. Live trapping for annual banding programs routinely occurs in the summer (July–August) during which waterfowl are trapped and fitted with leg bands inscribed with identifying numbers for various research and monitoring purposes [11]. The trapping methods used for annual banding programs allow for concurrent sample collection beneficial for IAV surveillance. Additionally, annual hunting season occurs during autumn migration and early winter (September–December), providing hunter-harvested birds for convenient sample collection [12]. However, this convenience-based sampling method has led to large gaps in IAV surveillance in wild waterfowl, particularly during spring migration (March–June), when access to birds is limited [10]. Nevertheless, it should be noted that IAV surveillance has occurred in wild waterfowl during the spring, but on a significantly smaller scale and mostly in shorebirds [13,14,15,16]. Consequently, active surveillance, though extensive, has not shown how IAV circulates through wild bird populations on a continuous timeline [17]. It is unclear how IAV persists in waterfowl populations, where it is persisting, or when and how various strains are circulating through the population. Current surveillance methods must be evaluated and adjusted to properly fill gaps in surveillance and better understand how the virus persists and circulates through the population.

Evidence suggests that, while IAVs are dispersed over a short period of time throughout migratory flyways, certain IAVs are maintained regionally, reappearing in the same locations each autumn migration, though the mechanisms behind this maintenance are not well understood [18]. Some studies have found that IAVs may survive in the environment, which can contribute to persistence within the waterfowl population. For example, laboratory studies have demonstrated the tenacity of IAVs and their ability to survive in water under optimal conditions [19,20,21]. Field-based studies have successfully isolated IAV from surface waters of waterfowl habitat and subsequently infected ducks, suggesting the potential for transmission and spread of IAV through water [22]. Understanding the circulation of IAVs in waterfowl year-round can aid in identifying mechanisms of viral persistence in both the waterfowl population and in their environment. 

The objective of this study was to fill the gap in IAV surveillance during spring migration to understand ecological and evolutionary dynamics of the virus in waterfowl during a historically under-sampled time of year. We can better understand persistence of IAV on a continuous timeline and provide insight into the mechanisms driving persistence by conducting year-round surveillance in one species of waterfowl at one location. We chose an important migratory stopover site for waterfowl with a rich history of IAV surveillance as the sampling location. We chose wild mallards, *Anas platyrhynchos*, as the study species as they are known to be readily infected by a genetically diverse range of IAVs, allowing them to represent IAVs circulating in the overall waterfowl population [2]. Here, we describe prevalence trends and provide phylogenetic analyses of IAVs from wild mallards in one location over two years to improve understanding of persistence of IAVs on a continuous timeline. 

## 2. Materials and Methods 

### 2.1. Sample Collection

Active IAV surveillance was conducted in wild mallards July 2017–July 2019 at an impounded coastal wetland complex located in the south–west Lake Erie basin near Port Clinton, Ohio, USA (41° 27’ 39.6’’ N, 82° 59’ 49.2’’ W). Cloacal swabs were collected under the Ohio Department of Natural Resources Scientific Collection Permits 19-120 and 20-281, US Fish and Wildlife Services Permit MB66162B-1, and The Ohio State University Institutional Animal Care and Use Committee protocol number 2007A0148 as previously described [17]. We collected waterfowl for swabbing via active trapping using swim-in and decoy traps (January–August) or via hunter-harvest during annual duck hunting season (September–December). Active-trapping was attempted during late winter (January–February) and spring migration (March–June), which is an IAV sample-collection method that has only minimally been utilized during this time of year previously. Trapping occurred nearly daily (weather dependent) Monday through Friday. The number of birds in the traps varied greatly each day throughout the study, ranging from zero birds to >40 birds. All individual mallards captured each day were swabbed. Swabbing of the same individuals on multiple days was possible. Sample collection during hunting season relied heavily on the number of hunters and their harvest each day, Sunday–Saturday. The number of samples varied greatly each day, ranging from zero birds to 50 birds, with Saturdays being the most productive. All individual mallards harvested by hunters each day were swabbed. Waterfowl population counts were visually estimated by wildlife biologists during 2018 and 2019 to assess the success of sample collection methods relative to population size.

### 2.2. Influenza A Virus Testing

RNA extraction was performed on all samples on the MagMAX Express 96 Magnetic Particle Processor (Applied Biosystems, Foster City, CA, USA; AM1836_DW_100_V2 program) using the Mag-Bind Viral DNA/RNA 96 Kit (Omega Bio-tek, Inc., Norcross, GA, USA) following a modified protocol. The modified protocol used 240 μL of TNA lysis buffer, 24 μL 17% sodium sulfite, 280 μL 100% isopropanol, 1.44 μL 50 mg/mL bovine serum albumin, 4 μL carrier RNA, 2 μL Xeno internal positive control template (VetMAX Xeno Internal Positive Control RNA, Life Technologies, Austin, TX, USA), 10 μL proteinase K, and 10 μL magnetic beads per reaction. Extraction included two washes with 400 μL VHB buffer and two washes with 500 μL SPR buffer. RNA was eluted into 50 μL nuclease-free water. Real-time reverse transcription polymerase chain reaction (rRT-PCR) targeting a segment of the IAV matrix gene was performed on all extracted RNA using SuperScript III One-Step RT-PCR System with Platinum Taq DNA Polymerase (ThermoFisher Scientific, Waltham, MA, USA) following an optimized protocol. The reaction mixture contained 15 μL 2× reaction buffer, 1 μL reverse transcriptase, 0.1 μL ROX reference dye (Life Technologies, Austin, TX, USA), 2.7 μL nuclease-free water, 0.5 μL of 6 μM M+64 probe [22], 0.5 μL of 20 μM M+25 forward primer [23], 0.5 μL of 20 μM M-124 reverse primer [23], 0.5 μL of 20 μM modified M-124 reverse primer [24], 1.2 μL of the Xeno internal positive control primer and probe mix (VetMAX Xeno Internal Positive Control–VIC Assay, Life Technologies, Austin, TX, USA), and 8 μL extracted RNA. Cycling conditions included 48 °C for 10 min, 95 °C for 10 min, and 40 cycles of 95 °C for 15 s and 60 °C for 45 s. Cycle threshold (Ct) values were calculated for each sample by setting the threshold at 5% of the positive control at cycle 40. Samples with a Ct <40 were considered positive. Viral isolation was attempted on all rRT-PCR positive samples and samples for which the internal positive control failed via inoculation into specific pathogen free embryonating chicken eggs using previously described methods [18]. All IAV isolates were submitted for whole genome sequencing at National Veterinary Services Laboratory (Ames, IA, USA). Additionally, RNA from rRT-PCR positive original samples with a Ct value <30 that were not successfully isolated were submitted for whole genome sequencing as part of the National Institutes of Health National Institute of Allergy and Infectious Disease (NIH-NIAID) Center of Excellence for Influenza Research and Surveillance Distributed Genomic Sequencing Cores program at University of Georgia (Athens, GA, USA) and Mount Sinai Medical School (New York, NY, USA) to add to the genomic data. Sequences were submitted to the NIH-NIAID Center of Excellence for Influenza Research and Surveillance Data Processing and Coordinating Center and subsequently submitted to GenBank (Table S1).

### 2.3. Phylogenetic Analysis

Phylogenetic analyses on nucleotide sequences were used to assess potential persistence and evolution of genetic lineages within a single location annually, with specific emphasis throughout spring migration. We constructed large-scale approximate maximum likelihood phylogenetic analyses on all eight gene segments with FastTree 2.1 using a general time reversible (GTR) substitution model [25] to identify monophyletic clades of interest that included viruses during spring migration. Maximum likelihood phylogenetic analyses were conducted on all nucleotide sequences of all six internal gene segments and the six HA and four NA gene segments obtained from samples collected during spring migration. We downloaded and included in the analyses additional IAV nucleotide sequences from avian host species in North America and Eurasia from the NIH-NIAID Influenza Research Database [26]. Sequences were aligned using MAFFT v7.308 [27] and were manually examined and trimmed to coding regions using MEGA-X v.10.1.7 [28]. We constructed time-scaled phylogenies for clades of interest with down-sampling. We evaluated time-stamped sequences using the Bayesian Markov chain Monte Carlo method with a GTR+Γ substitution model and lognormal relaxed clock executed in BEAST v1.10.4 [29]. Chains ran for at least 50 million generations and outputs were visualized in Tracer v1.7.1 to ensure effective sample size values >200. We completed and combined two independent runs were after removing a burn-in of 10% using LogCombiner v1.10.4 and were summarized into maximum clade credibility trees using Tree Annotator v1.10.4. All trees were visualized and edited in FigTree v1.4.4 and Adobe Illustrator (Adobe Inc., San Jose, CA, USA).

### 2.4. Statistical Analysis

95% confidence intervals were calculated for the proportion of positive birds using the standard error of the proportion in each season throughout the study period Logistic regressions were performed to determine association of age and sex with IAV infection. Odds ratios were generated between the predictors, age and sex, and the outcome, IAV. Significance was set to *p* < 0.05 (Stata special 87 edition 14.2, College Station, TX, USA).

## 3. Results

### 3.1. Sample Collection

**Figure 1:**
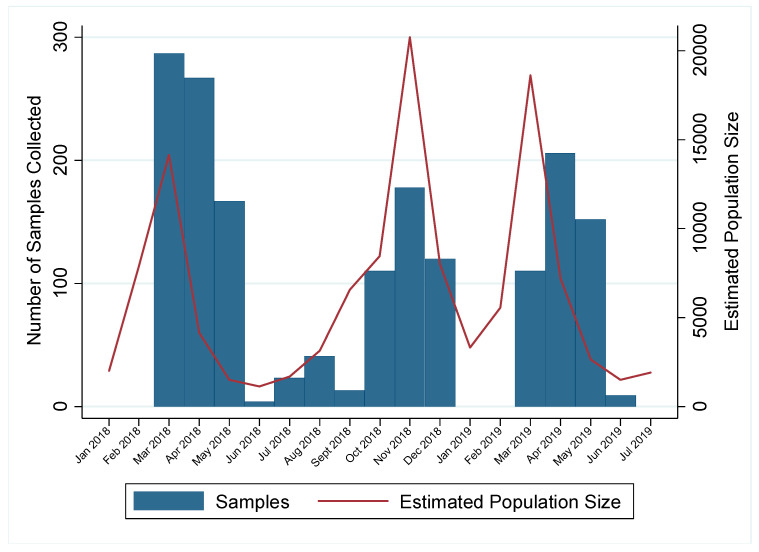
Number of samples collected for influenza A virus (IAV) surveillance in mallards and estimated population size of all waterfowl species near Port Clinton, Ohio, USA by month. Year-round IAV surveillance was conducted in wild mallards (*Anas platyrhynchos*) in one location for two years (July 20217–July 2019) to better capture the under-sampled spring migration. Samples for IAV surveillance collected from mallards by active trapping (January–August) and hunter harvest (September–December) are shown by blue bars. Estimated population size of waterfowl at the study location is shown by the red line. Data is shown as starting January 2018 as estimated population size was not available before that time.

Active trapping during the winter and spring months required extensive labor and effort and was most successful during times of high population size (Figure 1). Winter weather, particularly frozen marshes, reduced the success of active trapping due to low population size and the inability to set traps. Therefore, no samples were collected in January and February in either of the two years. Additionally, we hypothesize that low population size reduced the success of active trapping during the summers of 2018 and 2019, when active trapping has historically been fruitful (Figure 1). A total of 1178 samples were collected in spring months (March–June); 297 in summer months (June–August); 462 samples in autumn months (September–November); and 148 in winter months (December–February) over the two years. Though sample collection was limited at certain times due to weather and low population size, implementing active trapping during the spring yielded high sample numbers and provided prevalence estimates with high precision.

### 3.2. Prevelance

**Figure 2:**
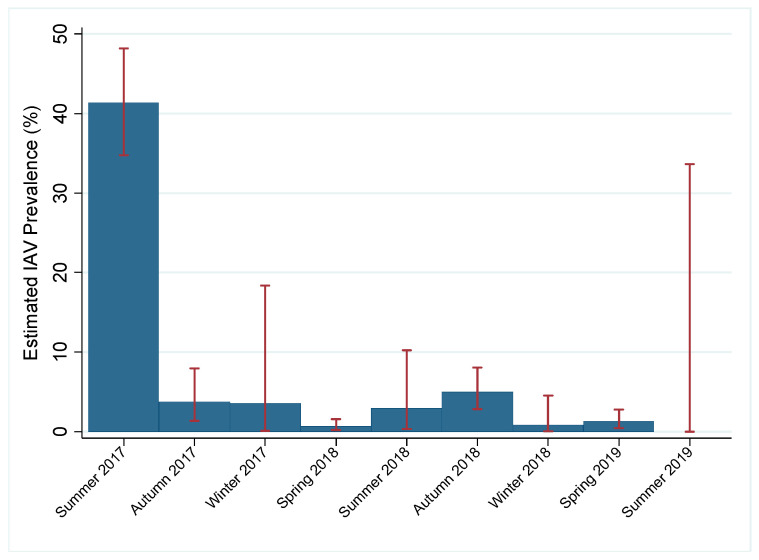
Estimated influenza A virus (IAV) prevalence in migratory mallards near Port Clinton, Ohio, USA by season. Year-round, active IAV surveillance in mallards (*Anas platyrhynchos*) was conducted for two years (July 2017–July 2019) in one location to fill a gap during spring migration and resulted in 2096 cloacal swabs. Viral isolation was attempted on all real-time reverse transcription polymerase chain reaction positive and undetermined samples. Prevalence was estimated by the proportion of viral isolates and the number of cloacal swabs collected during each season expressed as a percentage. Estimated IAV prevalence of mallards at the study location for each season of the study period is shown by blue bars with 95% confidence intervals shown by red error bars. Summer: June–August; autumn: September–November; winter: December–February; spring: March–May.

**Figure 3:**
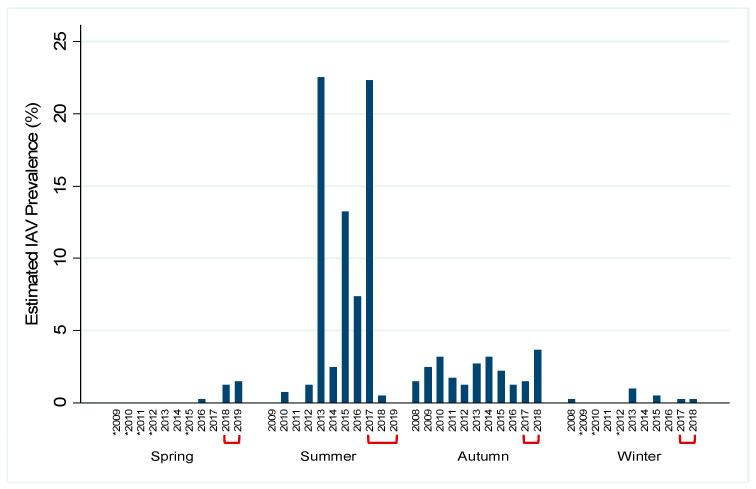
Historical influenza A virus (IAV) surveillance near Port Clinton, Ohio, USA by year and season. Year-round, Active IAV surveillance in wild mallards (*Anas platyrhynchos*) at one location was conducted from July 2017–July 2019 to represent the under-sampled spring season. Historical (autumn 2008–spring 2017) surveillance data for the study location is shown in order to demonstrate the gap in spring surveillance prior to the current study. IAV prevalence for each year by season was estimated by the proportion of IAV isolates and the number of cloacal swabs collected and is shown by the blue bars. Red brackets indicate the current study period. Asterisks (*) represent years for which no samples were collected during that season. Summer: June–August; autumn: September–November; winter: December–February; spring: March–May.

Of the 2096 samples, 267 (12.7%) tested positive for IAV by rRT-PCR while 130 had an internal positive control failure. Due to the high number of rRT-PCR internal control failures for samples during the study period and lack of historical rRT-PCR data, we estimated prevalence by virus isolation. The total estimated prevalence during the study period was 6.1% (95% confidence interval (CI): 0.050–0.071). Within a given year, estimated prevalence peaked in late summer and early autumn and decreased throughout the winter and spring months. Estimated prevalence was highest in summer months (31.3%, 95% CI: 0.260–0.366) and lowest during spring months (1.0%, 95% CI: 0.004–0.015; Figure 2). IAV subtypes varied throughout the study period; both pure and mixed subtypes were isolated (Table 1). Juvenile birds had 1.75 times the odds (*p* = 0.008) of having active IAV infection as compared to mature birds and high numbers of immune-naive juvenile birds at the end of breeding season. Additionally, females had 2.43 times the odds of having active IAV infection as compared to males (*p* < 0.0005). Historical data from active IAV surveillance in wild mallards at this location were compared to the current study period. Seasonal prevalence trends during the study period were comparable to past surveillance data, except for the summers of 2018 and 2019 when the estimated prevalence appeared lower than what has historically been observed (Figure 3). Additionally, the current study period estimated IAV prevalence during spring for which little data have been collected during past surveillance at the study location (Figure 3).

### 3.3. Phylogenetic Analysis

**Figure 4:**
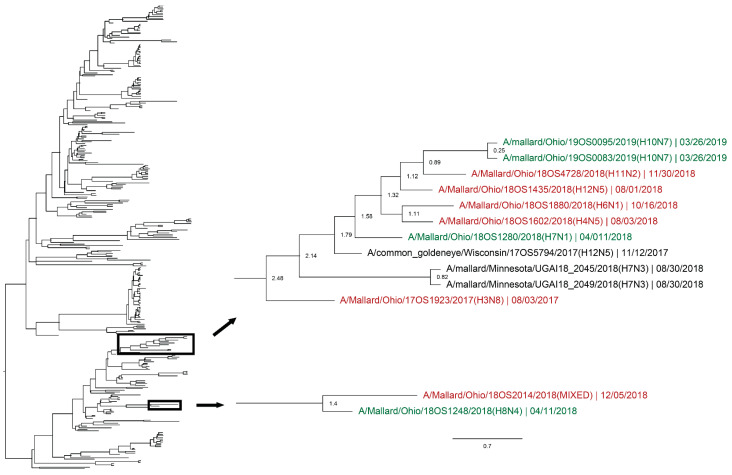
Maximum clade credibility tree of the PA gene segment. Active influenza A virus surveillance was conducted year-round in wild mallards (*Anas platyrhynchos*) in one location over two years. Time-stamped phylogenetic analysis with a general time reversible plus gamma substitution model revealed genetic persistence across multiple seasons, including the historically under-sampled spring season, in the relatively conserved PA gene segment. Highly supported clades (>0.95 posterior probability) containing viruses detected in the spring (green) who share a common ancestor with viruses detected in another season (red) throughout the study period with a time to most recent common ancestor of <2.5 years are highlighted. Node ages are indicated. Scale bar represents time in years.

**Figure 5:**
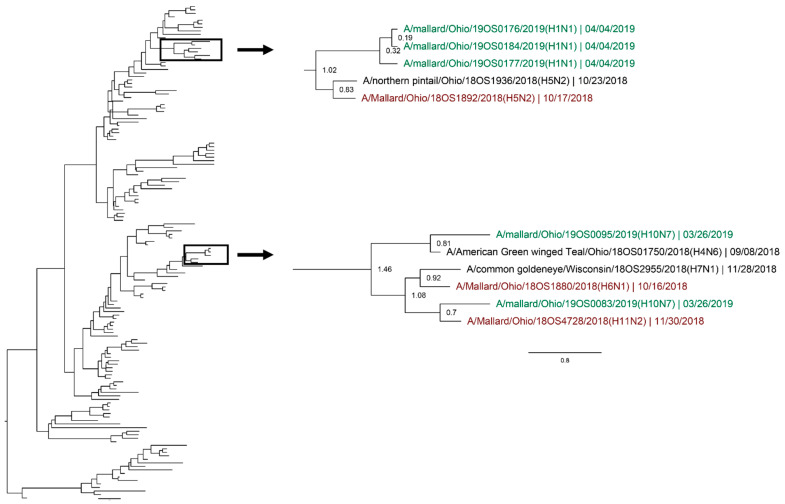
Maximum clade credibility tree of the M gene segment. Year-round, active influenza A virus surveillance was conducted in wild mallards (*Anas platyrhynchos*) in one location over two years. Time-stamped phylogenetic analysis with a general time reversible plus gamma substitution model revealed genetic persistence across multiple seasons, including the historically under-sampled spring season, in the relatively conserved M gene segment. Highly supported clades (>0.95 posterior probability) containing viruses detected in the spring (green) who share a common ancestor with viruses detected in another season (red) throughout the study period with a time to most recent common ancestor of <2.5 years are highlighted. Node ages are indicated. Scale bar represents time in years.

**Figure 6:**
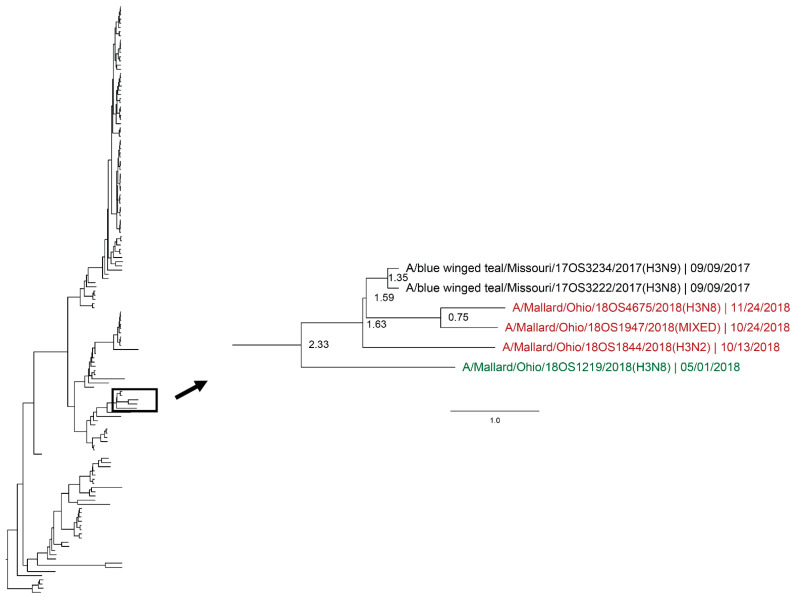
Maximum clade credibility tree of the H3 gene segment. Year-round, active influenza A virus surveillance was conducted in wild mallards (*Anas platyrhynchos*) in one location over two years. Time-stamped phylogenetic analysis with a general time reversible plus gamma substitution model revealed genetic persistence across multiple seasons, including the historically under-sampled spring season, in the HA gene segment. Highly supported clades (>0.95 posterior probability) containing viruses detected in the spring (green) who share a common ancestor with viruses detected in another season (red) throughout the study period with a time to most recent common ancestor of <2.5 years are highlighted. Node ages are indicated. Scale bar represents time in years.

**Figure 7:**
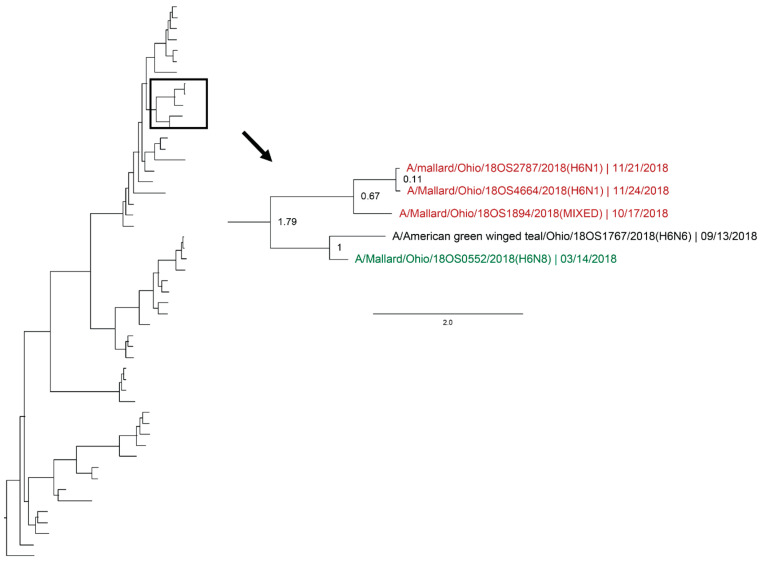
Maximum clade credibility tree of the H6 gene segment. Year-round, active influenza A virus surveillance was conducted in wild mallards (*Anas platyrhynchos*) in one location over two years. Time-stamped phylogenetic analysis with a general time reversible plus gamma substitution model revealed genetic persistence across multiple seasons, including the historically under-sampled spring season, in the HA segment. Highly supported clades (>0.95 posterior probability) containing viruses detected in the spring (green) who share a common ancestor with viruses detected in another season (red) throughout the study period with a time to most recent common ancestor of <2.5 years are highlighted. Node ages are indicated. Scale bar represents time in years.

Whole genomic sequences were obtained from 127 viral isolates and one original sample (sequenced RNA from original sample swab) and were used for phylogenetic analysis. Analysis revealed instances of local genetic lineages of several gene segments that persisted throughout the spring season. Clades that included viruses detected in the spring and viruses detected in another season at the study location who share an estimated time to most recent common ancestor (TMRCA) of <2.5 years and have a posterior probability of >0.95 were considered to show genetic persistence and evolution throughout the study period. All viruses (*n* = 11) recovered during the spring season uncovered evidence of genetic persistence across multiple seasons in at least one gene segment. Analysis suggests local evolution of some highly genetically conserved internal gene segments of IAV. A phylogeny of the PA segment shows a small monophyletic clade that includes IAVs detected in mallards at the study location in summer of 2017, spring, summer, and autumn of 2018, and spring of 2019. The estimated TMRCA of this clade was 2.48 years (95% highest posterior density (HPD): 2.18–2.76; Figure 4). Additionally, the PA segment of a virus detected in spring of 2018 is sister taxa to the PA segment of a virus detected in autumn of 2018 with an estimated TMRCA of 1.4 (95% HPD: 1.17–1.73; Figure 4). Analysis of the M segment reveals two clades with high support that includes IAVs isolated during autumn migration and the proceeding spring migration (Figure 5). The estimated TMRCA of those clades were both less than 1.5 years, representing close relation between the viruses isolated during autumn and spring migration. Analysis of the antigenic gene segments also indicates instances of close relation between viruses isolated during spring migration and viruses isolated during the proceeding autumn migration. Both the H3 and H6 gene segments consist of clades with high support that include IAVs from spring and the following autumn migration of 2018. The TMRCA of those clades is 2.33 years (95% HPD: 1.76–3.02; Figure 6) and 1.79 years (95% HPD: 1.06–2.52; Figure 7), respectively. Analysis of the PB2, PB1, H10, NP, and NS gene segments also showed evidence of local genetic persistence under the chosen parameters (Figures S1–S6).

## 4. Discussion

Understanding the ecology and epidemiology of IAV in wild waterfowl is pertinent for the protection of animal and public health [1]. Active IAV surveillance in waterfowl is imperative to this understanding. Extensive active surveillance in waterfowl has provided insight into prevalence trends and transmission dynamics of IAV in this population. However, convenience-based sampling approaches have led to gaps in the overall understanding of IAV in wild waterfowl [2,10]. In particular, spring has remained an under-sampled season, particularly at the study location, when access to birds is limited due to lack of sampling methods [17].

Though active trapping for surveillance is expensive and labor-intensive [30], we have shown that this collection method can be used effectively during spring migration, a time when active trapping has not traditionally been employed. However, success of trapping, measured by the number of birds trapped, seems to be dependent on optimal conditions, including population size and weather conditions, as trapping during the late winter months was unproductive. Successful trapping was associated with the birds’ annual cycle; it was most effective with the influx of birds during peak spring migration and decreased in productiveness as breeding season began in early summer. Output of trapping then increased as breeding season closed in late summer when females leave nests and hatchlings enter the population. Though the number of birds captured may be dependent on population size and it may only be productive at certain times of the year, implementation of active trapping during times other than summer is a useful tool for active IAV surveillance in wild waterfowl.

Spring surveillance conducted during the study period has provided insight on prevalence trends during times of year where historical data is limited. Historical surveillance found prevalence of IAV in the wild waterfowl population in North America peaks as high at 60% during late summer and early autumn as breeding season ends, and then falls to approximately 2% during south-bound autumn migration [1,2,31]. In our study, there was some deviation from the expected dramatic peak in prevalence during the summers of 2018 and 2019, which we hypothesize is due to lower than normal sample numbers. Historically, active trapping has yielded >200 samples from mallards during the summer season at the study location, with upwards of 20% IAV positive. During the summer of 2018, lower than usual population numbers at the study location are believed to have had an effect on trapping success, leading to <70 samples, and viral recovery. Additionally, the study period ended in the middle of July of summer 2019, which is earlier than the typical influx of juveniles, also reducing sample numbers. Furthermore, limited surveillance has shown that IAV prevalence continues to fall during north-bound spring migration, to as low as 0.25%, before rising again near the end of breeding season [2]. Increased sampling throughout the study period, particularly during north-bound migration, led us to estimate IAV prevalence in the waterfowl population to be slightly higher, around 1%, during this time. However, this could be specific to the study location, a popular stopover site for many ducks, where large congregations of waterfowl create conditions conducive to viral spread and may not be reflective of the overall waterfowl population. Additionally, use of viral isolation for prevalence estimates may be biased by our choice of culture system and may be deflated when compared to estimates by rRT-PCR [32]. Furthermore, without a banding/marking system, active trapping may have led to the recapture/re-swabbing of some individuals. If individuals had active IAV infection during multiple capture events, this may have skewed prevalence estimates. However, it is important to note that IAV is present in the population during every season, even during north-bound spring migration. Though cold temperatures and low population numbers made sampling difficult in late winter months (January–February), this project provided an initial step towards improving understanding of IAV persistence between autumn migration and the summer breeding season.

Differences in IAV infection rates among ages and sexes of waterfowl have often been detected during routine IAV surveillance. During our study period, juvenile ducks had increased odds of having active IAV infection compared to mature ducks. This is consistent with what has been described previously, where increased rates of IAV infection in juvenile wild waterfowl are believed to be due to a lack of protective immunity gained through prior exposures [33,34,35]. Additionally, we found female mallards to have increased odds of active IAV infection compared to male ducks. Though results of studies assessing differences in active IAV infection is sexes are variable, our results are consistent with previous research in mallards which found higher IAV prevalence in females [32]. However, considering the lack of consistent findings comparing IAV infection between sexes in waterfowl, it is unclear what would cause increased infection in female and more research is warranted.

Although use of active trapping for sample collection during spring migration required extensive effort and was most productive during peak migration, the 11 IAVs isolated during this time provide important genetic information about the virus during a previously under-sampled time. Phylogenetic analyses revealed genetic lineages of IAVs at the sample location that persist over time that include all 11 viruses recovered during spring. Previous surveillance has shown persistence of genetically similar IAVs at several migratory stopover sites throughout the Mississippi Flyway during each autumn migration [18]. Additionally, it has been suggested that viruses from autumn migration may seed the prevalence peak in summer [36]. Analysis of the viruses isolated from mallards throughout the study period revealed IAVs recovered during spring migration were closely related to IAVs isolated during preceding and/or proceeding autumn migration and summer seasons. Interestingly, no IAV subtype was isolated in all four seasons; a large range of subtypes, both pure and mixed, were isolated throughout the study period. However, this is not surprising due to IAV’s antigenic properties and the ability of the virus to reassort. Nonetheless, phylogenetic analysis revealed genetic persistence in both the antigenic and internal gene segments. Though isolation of genetically similar viruses from migratory mallards in successive seasons does not solve the issue of viral persistence and whether that persistence occurs within the waterfowl population or with the added interaction of the environment, it does suggest that IAVs can persist and evolve within a single location over successive seasons, throughout the annual cycle of waterfowl. Many factors, such as water, sediment, and other organisms, have been hypothesized to play a role in maintenance of IAV in the environment and therefore the wild bird population during the overwintering and spring period [21,37,38,39,40]. However, these mechanisms are mostly investigated as in vitro studies performed in the laboratory or are experimental in nature with limited field-based evidence. The evidence of genetic lineages of IAVs at one migratory stopover site provided by this study suggests the possibility that IAVs may be able to persist in waterfowl alone and without the involvement of environmental factors or other organisms.

Continued active IAV surveillance and research in waterfowl is essential for the protection of animal and public health. It is imperative to wholly understand IAV in its natural reservoir in order to make science-based decisions and recommendations that reduce the risk to animals and humans. However, the consistent isolation of highly genetically similar IAVs over time at one location has implications for future IAV surveillance in waterfowl. Cross-sectional studies make up a large proportion of IAV surveillance methods in wild birds, where samples are collected at the same location at the same time year after year [41,42,43]. If the goal of IAV surveillance is to capture the genetic diversity in order to increase outbreak and pandemic preparedness [44], conducting surveillance in the same location year after year will result in IAVs that are highly similar and, as a consequence, will provide limited knowledge of the genetic diversity of IAV in the entire wild waterfowl population. Therefore, it is imperative that future IAV surveillance conducted in wild waterfowl take this into account. It is essential that future surveillance methods be conducted in a way that aims to answer specific questions. Using methodology informed by hypothesis driven questions will allow scientists to provide the most valuable data and ultimately help reduce the risk of IAV to animal and public health.

## Figures and Tables

**Table 1:** Influenza A virus (IAV) subtypes recovered from wild mallards near Port Clinton, Ohio, USA by season. Active IAV surveillance in wild mallards (*Anas platyrhynchos*) was conducted year-round July 2017–July 2019 in an attempt to fill a gap in surveillance during the spring season. IAV subtypes isolated from mallards are shown by season. Both pure and mixed subtypes were isolated throughout the study period.

		Season			
		Spring	Summer	Autumn	Winter
**Pure Subtypes**	**H1N1**	3	5	1	
	**H1N2**		1	1	
	**H1N8**		12		
	**H3N1**			1	
	**H3N2**		10	1	
	**H3N8**	1	51	1	
	**H4N5**		1		
	**H4N6**			4	
	**H5N2**			2	
	**H6N1**			4	
	**H6N8**	1			
	**H7N1**	1			
	**H8N4**	1			
	**H10N1**		2		
	**H10N4**	1			
	**H10N7**	3	1		1
	**H10N8**		2		
	**H11N2**			1	
	**H11N3**			2	
	**H11N9**			1	
	**H12N5**		1		
**Mixed Subtypes**	**H1N1,3**				1
	**H1,3N2,8**		1		
	**H1,3N8**		1		
	**H3N1,8**		2		
	**H3,4N2,8**		1		
	**H3,4N3,6,8**		1		
	**H3,4N8**			1	
	**H3,5N2,8**		1		
	**H6,11N1,9**			1	
	**TOTAL**	11	93	21	2

## Supplementary Materials

The following are available online at https://www.mdpi.com/1999-4915/12/6/632/s1, Figure S1: Maximum clade credibility trees of the PB2 gene segment, Figure S2: Maximum clade credibility trees of the PB1 gene segment, Figure S3: Maximum clade credibility trees of the H10 gene segment, Figure S4: Maximum clade credibility trees of the NS gene segment, Figure S5: Maximum clade credibility trees of the NP gene segment, Figure S6: Maximum clade credibility trees of the N8 gene segment, Table S1: GenBank accession numbers of viral isolates recovered from mallards during the study period.
