# Supplementary material for: Year-Round Influenza a Virus Surveillance in Mallards (Anas platyrhynchos) Reveals Genetic Persistence During the Under-Sampled Spring Season
Source: Viruses. 2020 Jun 11;12(6):632. doi: 10.3390/v12060632 (PMC7354581; doi:10.3390/v12060632)
Supplement: Supplementary file 1 [file viruses-12-00632-s001.zip › Figures S1-S6.pdf]

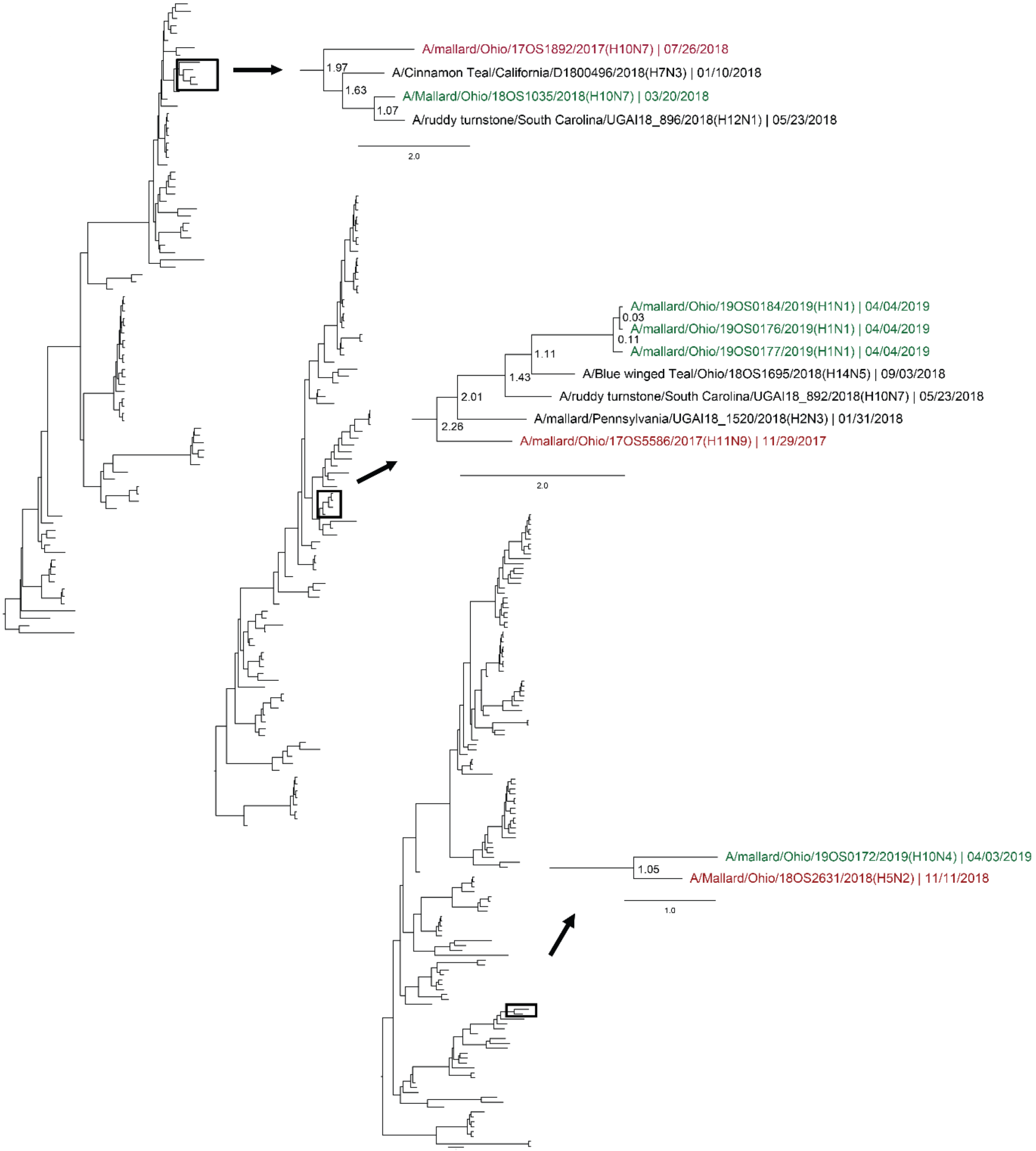

Figure S1: Maximum clade credibility trees of the PB2 gene segment. Year-round, active influenza A virus surveillance was conducted in wild mallards (*Anas platyrhynchos*) in one location over two years. Time-stamped phylogenetic analysis with GTR+Γ substitution model revealed genetic persistence across multiple seasons, including the historically under-sampled spring season. Highly supported clades (>0.95 posterior probability) containing viruses detected in the spring (green) who share a common ancestor with viruses detected in another season (red) throughout the study period with a TMRCA of <2.5 years are highlighted. Node ages are indicated. Scale bar represents time in years.

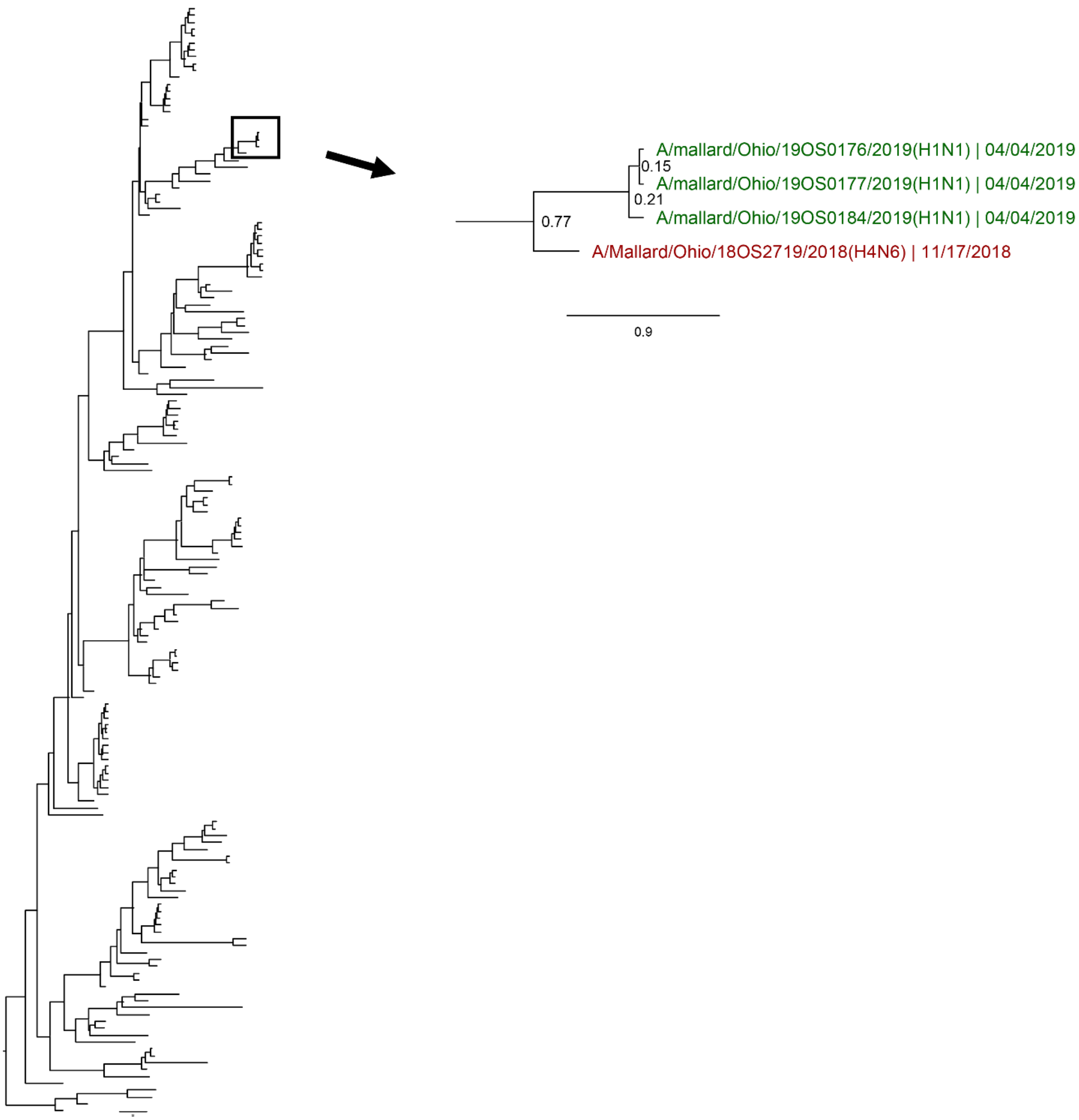

Figure S2: Maximum clade credibility trees of the PB1 gene segment. Year-round, active influenza A virus surveillance was conducted in wild mallards (*Anas platyrhynchos*) in one location over two years. Time-stamped phylogenetic analysis with GTR+Γ substitution model revealed genetic persistence across multiple seasons, including the historically under-sampled spring season. Highly supported clades (>0.95 posterior probability) containing viruses detected in the spring (green) who share a common ancestor with viruses detected in another season (red) throughout the study period with a TMRCA of <2.5 years are highlighted. Node ages are indicated. Scale bar represents time in years.

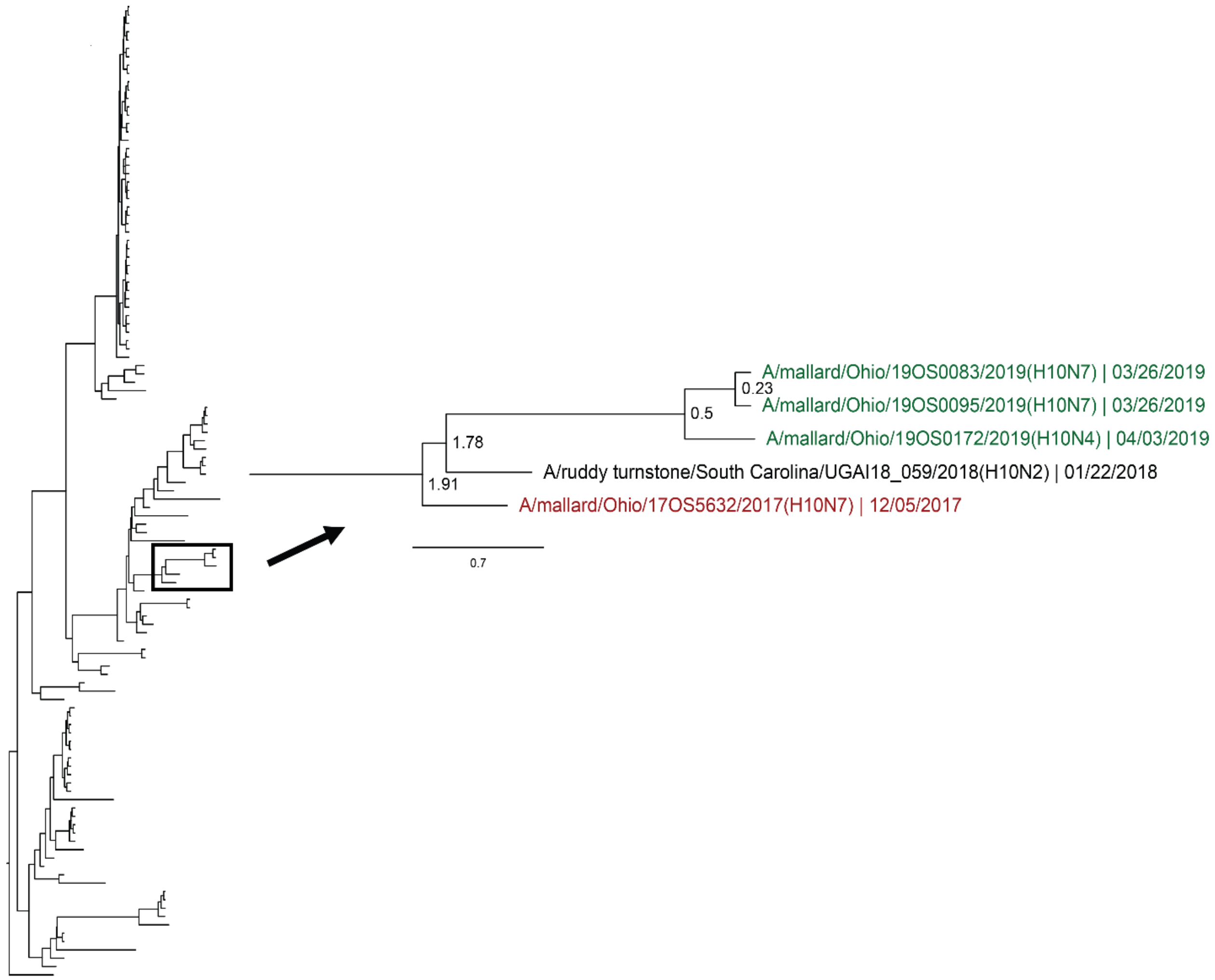

Figure S3: Maximum clade credibility trees of the H10 gene segment. Year-round, active influenza A virus surveillance was conducted in wild mallards (*Anas platyrhynchos*) in one location over two years. Time-stamped phylogenetic analysis with GTR+Γ substitution model revealed genetic persistence across multiple seasons, including the historically under-sampled spring season. Highly supported clades (>0.95 posterior probability) containing viruses detected in the spring (green) who share a common ancestor with viruses detected in another season (red) throughout the study period with a TMRCA of <2.5 years are highlighted. Node ages are indicated. Scale bar represents time in years.

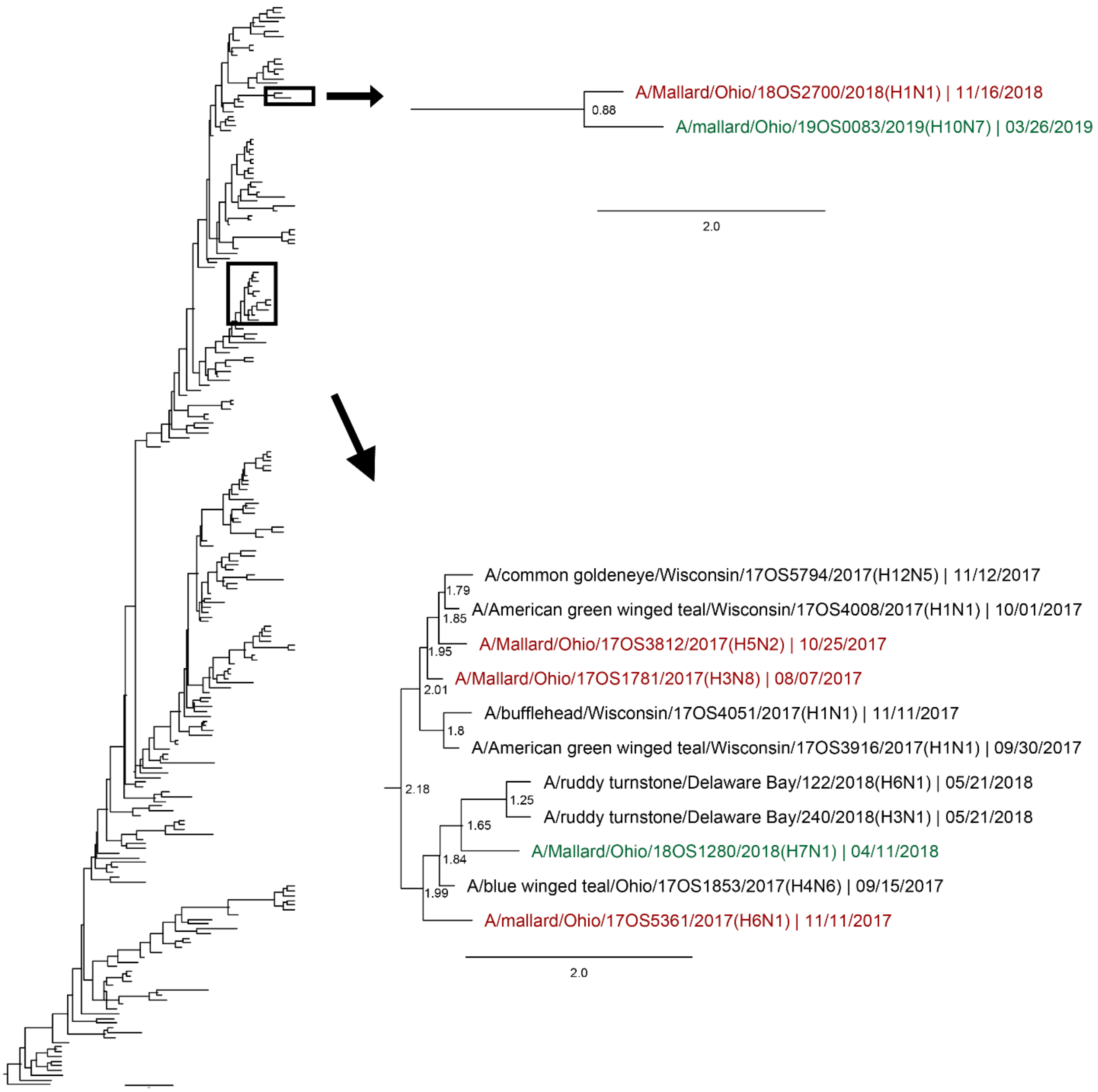

Figure S4: Maximum clade credibility trees of the NS gene segment. Year-round, active influenza A virus surveillance was conducted in wild mallards (*Anas platyrhynchos*) in one location over two years. Time-stamped phylogenetic analysis with GTR+Γ substitution model revealed genetic persistence across multiple seasons, including the historically under-sampled spring season. Highly supported clades (>0.95 posterior probability) containing viruses detected in the spring (green) who share a common ancestor with viruses detected in another season (red) throughout the study period with a TMRCA of <2.5 years are highlighted. Node ages are indicated. Scale bar represents time in years.

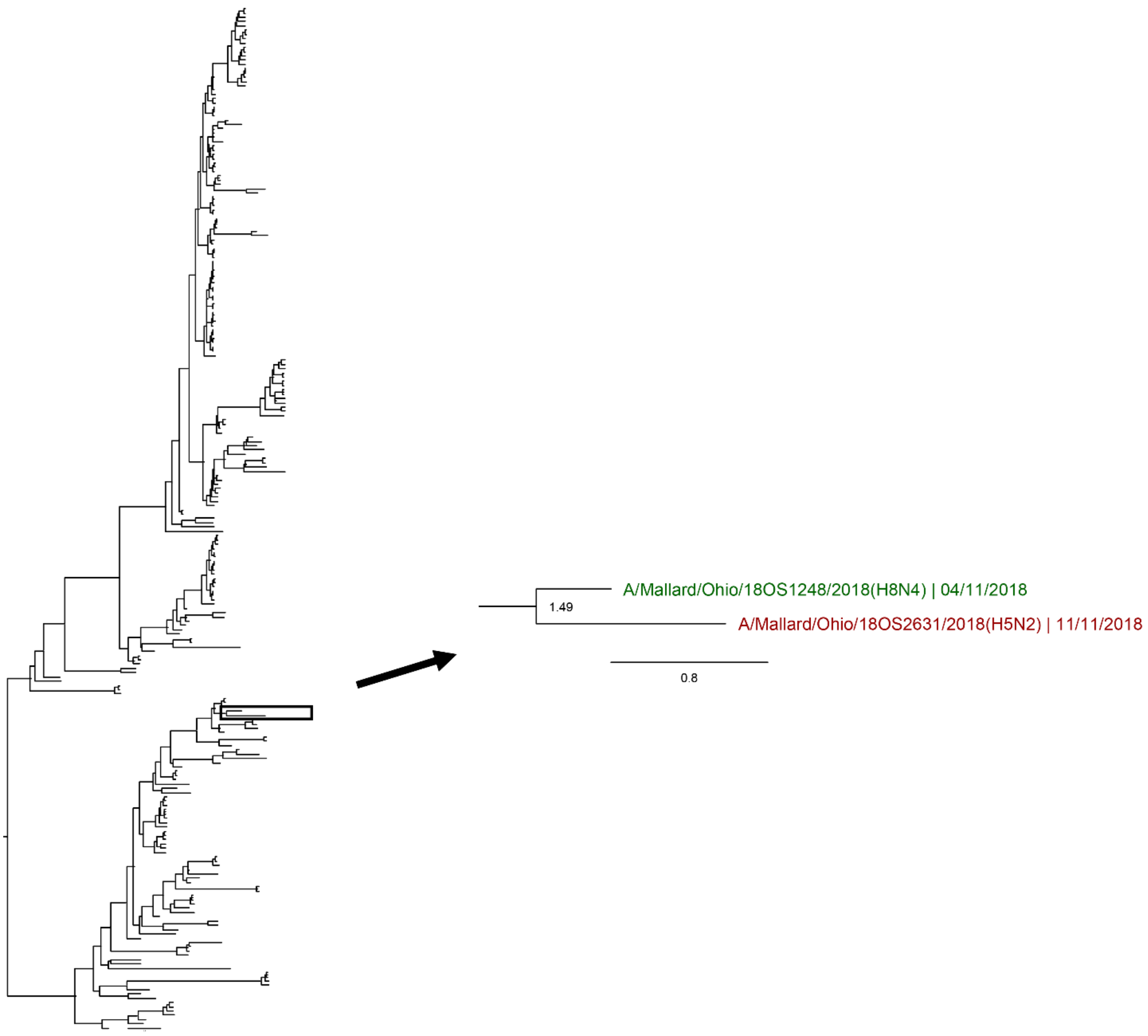

Figure S5: Maximum clade credibility trees of the NP gene segment. Year-round, active influenza A virus surveillance was conducted in wild mallards (*Anas platyrhynchos*) in one location over two years. Time-stamped phylogenetic analysis with GTR+Γ substitution model revealed genetic persistence across multiple seasons, including the historically under-sampled spring season. Highly supported clades (>0.95 posterior probability) containing viruses detected in the spring (green) who share a common ancestor with viruses detected in another season (red) throughout the study period with a TMRCA of <2.5 years are highlighted. Node ages are indicated. Scale bar represents time in years.

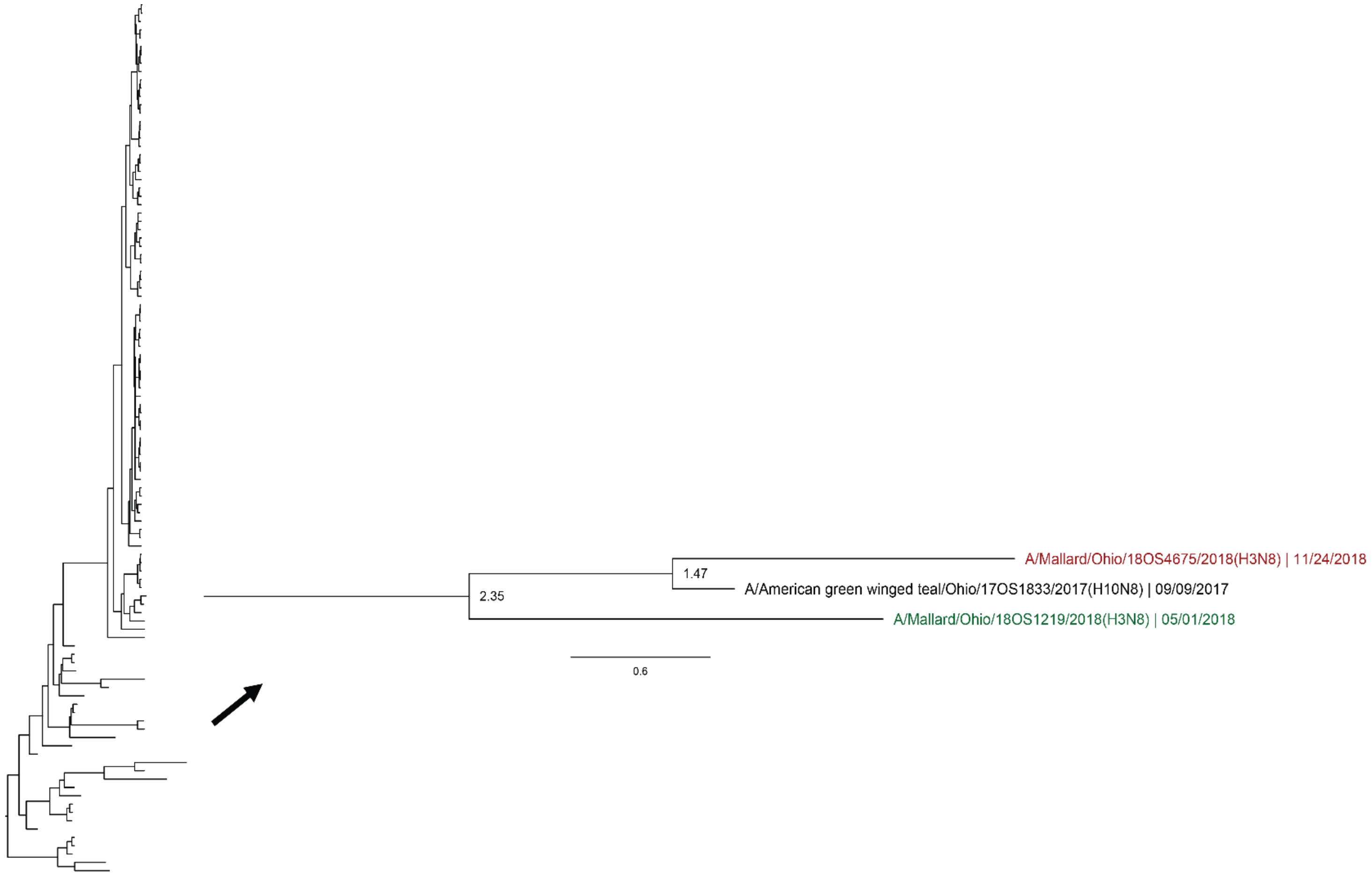

Figure S6: Maximum clade credibility trees of the N8 gene segment. Year-round, active influenza A virus surveillance was conducted in wild mallards (*Anas platyrhynchos*) in one location over two years. Time-stamped phylogenetic analysis with GTR+Γ substitution model revealed genetic persistence across multiple seasons, including the historically under-sampled spring season. Highly supported clades (>0.95 posterior probability) containing viruses detected in the spring (green) who share a common ancestor with viruses detected in another season (red) throughout the study period with a TMRCA of <2.5 years are highlighted. Node ages are indicated. Scale bar represents time in years.
